# Complete Locked-in and Locked-in Patients: Command Following Assessment and Communication with Vibro-Tactile P300 and Motor Imagery Brain-Computer Interface Tools

**DOI:** 10.3389/fnins.2017.00251

**Published:** 2017-05-05

**Authors:** Christoph Guger, Rossella Spataro, Brendan Z. Allison, Alexander Heilinger, Rupert Ortner, Woosang Cho, Vincenzo La Bella

**Affiliations:** ^1^Guger Technologies OGGraz, Austria; ^2^g.tec Medical Engineering GmbHSchiedlberg, Austria; ^3^ALS Clinical Research Center, Biomedicina e Neuroscienze Cliniche (BioNeC), University of PalermoPalermo, Italy

**Keywords:** communication, ALS, BCI, EP, vibro-tactile, motor imagery

## Abstract

Many patients with locked-in syndrome (LIS) or complete locked-in syndrome (CLIS) also need brain-computer interface (BCI) platforms that do not rely on visual stimuli and are easy to use. We investigate command following and communication functions of mindBEAGLE with 9 LIS, 3 CLIS patients and three healthy controls. This tests were done with vibro-tactile stimulation with 2 or 3 stimulators (VT2 and VT3 mode) and with motor imagery (MI) paradigms. In VT2 the stimulators are fixed on the left and right wrist and the participant has the task to count the stimuli on the target hand in order to elicit a P300 response. In VT3 mode an additional stimulator is placed as a distractor on the shoulder and the participant is counting stimuli either on the right or left hand. In motor imagery mode the participant is instructed to imagine left or right hand movement. VT3 and MI also allow the participant to answer yes and no questions. Healthy controls achieved a mean assessment accuracy of 100% in VT2, 93% in VT3, and 73% in MI modes. They were able to communicate with VT3 (86.7%) and MI (83.3%) after 2 training runs. The patients achieved a mean accuracy of 76.6% in VT2, 63.1% in VT3, and 58.2% in MI modes after 1–2 training runs. 9 out of 12 LIS patients could communicate by using the vibro-tactile P300 paradigms (answered on average 8 out of 10 questions correctly) and 3 out of 12 could communicate with the motor imagery paradigm (answered correctly 4,7 out of 5 questions). 2 out of the 3 CLIS patients could use the system to communicate with VT3 (90 and 70% accuracy). The results show that paradigms based on non-visual evoked potentials and motor imagery can be effective for these users. It is also the first study that showed EEG-based BCI communication with CLIS patients and was able to bring 9 out of 12 patients to communicate with higher accuracies than reported before. More importantly this was achieved within less than 15–20 min.

## Introduction

**Figure 1:**
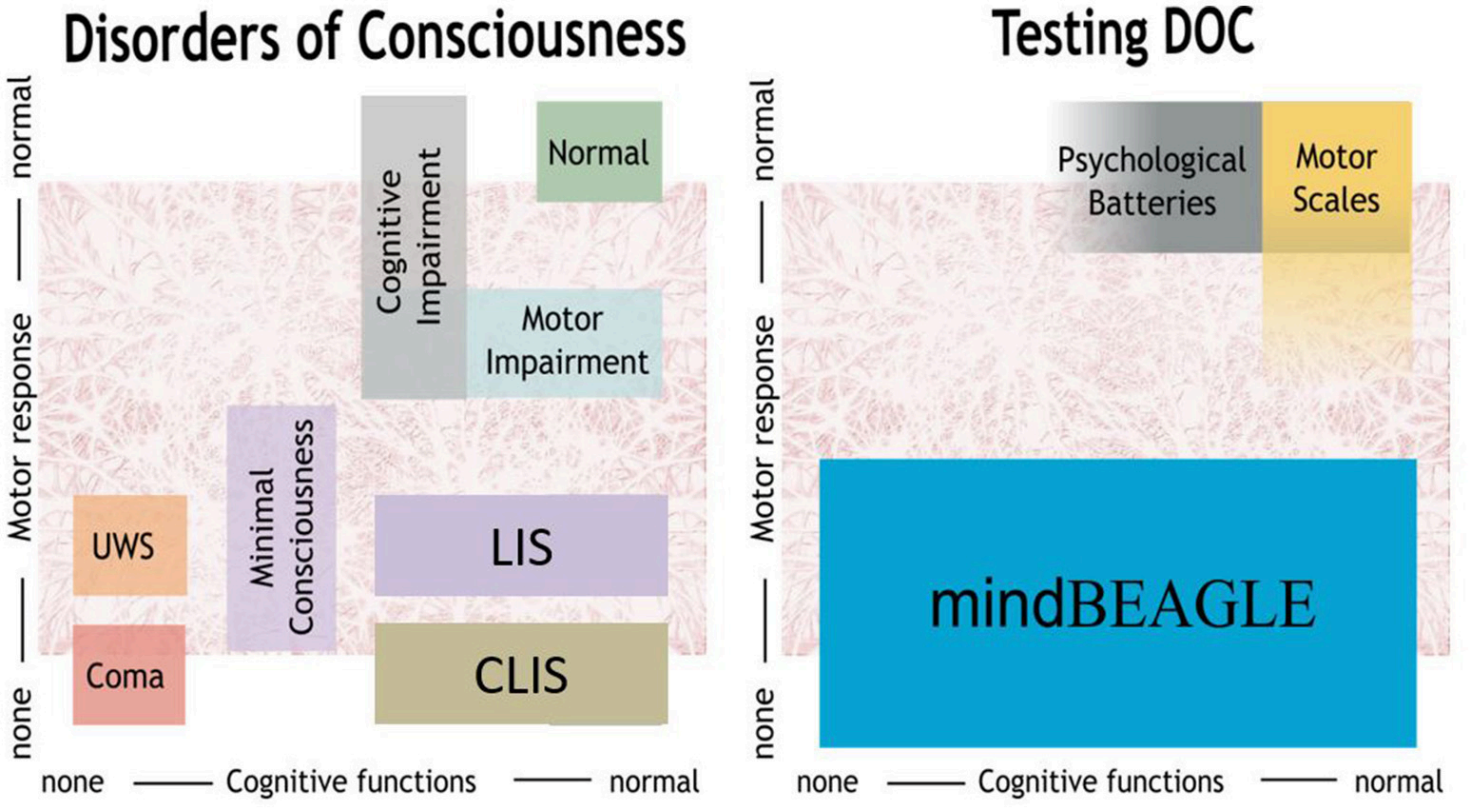
**mindBEAGLE provides an assessment battery for patients with disorders of consciousness such as unresponsive wakefulness state (UWS) or minimally conscious state (MCS), as well as locked-in syndrome (LIS) and complete locked-in syndrome (CLIS)**.

The left panel of Figure 1 categorizes different types of persons based on their available cognitive and motor functions. Coma patients do not exhibit cognitive or motor functions, and patients in the unresponsive wakefulness state (UWS) presumably have very limited or no motor and cognitive functions. Minimal consciousness (MCS) patients also do not have reliable voluntary motor control, and seem to have substantial cognitive impairment, although their cognitive function may fluctuate. These three types of patients are typically categorized as having a disorder of consciousness (DOC). Locked-in (LIS) and completely locked-in (CLIS) patients show limited or no motor response, and assessing cognitive function can thus also be difficult. LIS describes a clinical status of quadriplegia and anarthria, with preserved eye gaze movements. In CLIS, there is also oculomotor impairment and no communication is possible by any mean. LIS and CLIS patients may have healthy cognitive function, or may exhibit substantial impairment (Kübler et al., 2001).

To investigate remaining cognitive functions in patients with DOC, experts rely primarily on tests that require voluntary motor control, such as the Glasgow Coma Scale (GCS) or the Coma Recovery Scale—Revised (CRS-R) (Monti et al., 2010; Giacino et al., 2012; Risetti et al., 2013; Gibson et al., 2014). However, recent work has capitalized on brain-computer interface (BCI) systems, which typically provide communication or device control via direct measures of brain activity (Wolpaw et al., 2002; Wolpaw and Wolpaw, 2012). Since EEG BCIs can assess mental activity and even enable communication without requiring movement, some groups have extended EEG BCIs to assessment and communication for persons with DOC (Risetti et al., 2013; Guger et al., 2016b; Ortner et al., in press). The EEG-based mindBEAGLE BCI system was recently developed to provide a convenient, fast, and practical platform for cognitive assessment of command following and communication system for such patients (Ortner et al., 2013, in press; Guger et al., 2016b). This system relies on P300 and motor imagery BCIs and a step-by-step explanation is given in (Ortner et al., in press).

EEG-based BCIs can utilize different approaches, such as: (i) transient evoked potentials (like the N200 or P300) (Guger et al., 2009, 2016a; Lugo et al., 2014), (ii) steady-state evoked potentials (visual or somatosensory) (Bin et al., 2009; Ahn et al., 2016), or (iii) motor imagery (Guger et al., 2003; Acqualagna et al., 2016). P300 BCIs are often used for spelling applications, and were tested with LIS patients in several studies (Fazel-Rezai et al., 2012). These systems typically flash different characters on the screen. By silently counting each time a target character flashes, users can produce a P300 to target flashes only, and the BCI can use this signal to infer user intent. Group studies showed classification accuracies of about 90% for 81 subjects with a black and white speller, and the newer face speller has led to accuracy as high as 100% for 17 subjects (Guger et al., 2009, 2016a; Kaufmann et al., 2011). P300 BCIs based on auditory (Risetti et al., 2013; Rutkowski, 2016) or vibrotactile stimuli have also been validated (Lugo et al., 2014; Gibson et al., 2016). Both auditory and vibrotactile P300 BCIs typically have a smaller vocabulary and lower accuracy than their visual counterparts. However, they can help patients who cannot see, and vibrotactile BCIs can be used in loud environments. In previous work using vibrotactile P300 BCIs for LIS patients, 6 LIS patients attained a mean accuracy of 80% in a paradigm with 2 tactile stimulators (left and right hand) and 55.3% in a paradigm with 3 tactile stimulators (left and right hand, neck). In both paradigms, chance accuracy was 12.5%, and the results were statistically significant (Lugo et al., 2014). Recently also a system using functional near infrared spectroscopy was used for communication with CLIS patients and patients entering CLIS in more than 40 sessions (Chaudhary et al., 2017). Up to now EEG-based communication with CLIS was not shown (Chaudhary et al., 2017).

Motor imagery BCIs usually instruct the user to imagine a left hand or right hand movement to produce event-related (de-)synchronization (ERD/S) in the alpha and beta frequency ranges over the sensorimotor cortex (Guger et al., 2003; Pfurtscheller et al., 2010; Acqualagna et al., 2016). In a group study, 20 healthy people attained a mean accuracy of 80.7% after 20 min of training (Ortner et al., 2015). Motor imagery BCIs have also been validated with LIS patients (Kübler et al., 2005; McFarland and Vaughan, 2016). Motor imagery paradigms were also used in 16 UWS patients, and 3 of them were able to control the BCI system with an accuracy above chance level (Cruse et al., 2011).

Amyotrophic Lateral Sclerosis (ALS) is a well-known cause of LIS and then CLIS. Patients with ALS, particularly in its later stages, are more likely to exhibit sleep-wake disturbances than healthy controls (Lo Coco et al., 2011). Thus, they may have fluctuating periods of conscious awareness that are not easy to identify (since their capacity for voluntary movement is reduced or absent), confounded by the aforementioned possibility of reduced overall cognitive capacity. These patients may also have ocular impairments that could limit their use of eye-tracking devices or other assistive communication tools that require a healthy visual system (Spataro et al., 2014). These factors suggest that persons with LIS or CLIS, like persons with DOC, may benefit from a system designed to assess conscious awareness and provide communication for persons who can neither move nor see.

The current study aimed to evaluate mindBEAGLE's P300 and motor imagery paradigms for assessment of cognitive function and communication for LIS patients. Assessment in this context means to test if a person can follow commands and is able to perform a paradigm. We also sought to compare these two approaches to each other. P300 based systems typically need less training time and achieve higher accuracies than motor imagery based BCI systems (Guger et al., 2003, 2009; Allison and Neuper, 2010; Acqualagna et al., 2016), which suggests that P300 BCIs may be more practical for patients who do not have time for lengthy training. We also conducted sham runs with healthy controls to verify system performance. EEG-based BCI systems were not successfully used with CLIS patients so far and therefore it is tested if we can establish communication with them.

## Methods

### Hardware and software

The mindBEAGLE system provided the hardware and software platform for all recording, stimulus presentation, and real-time data analysis. Each system includes a laptop with installed software, three vibrotactile stimulators, two in-ear headphones, one g. USBamp biosignal amplifier with 16 channels and 24 Bit ADC resolution, and one EEG cap with 16 g. LADYbird active EEG electrodes. The EEG is sampled at 256 Hz and filtered between 0.1 and 30 Hz. Data were recorded from Fz, C3, Cz, C4, CP1, CPz, CP2, Pz for the P300 paradigms, and the motor imagery paradigm used sites FC3, FC4, C5, C1, C2, C6, CP3, and CP4 as well.

#### Assessment

This study presents three of mindBEAGLE's assessment paradigms:

1. Vibro-tactile stimulation with 2 tactors (VT2): During this paradigm, the left and right wrists are randomly stimulated with a vibro-tactile stimulator for 100 ms each. One stimulator delivers 87.5% of the stimuli, and the other stimulator presents only 12.5% of the stimuli. The subject is verbally instructed to count silently the stimuli on the hand that receives the less probable target stimuli, which is called the target hand. During each run, the subject performs this task four times, with the target hand selected randomly each time, which results in a recording time of 2.5 min.
2. Vibro-tactile stimulation with 3 tactors (VT3): During this paradigm, in addition to tactors on the left and right hands, one tactor is placed to the back or shoulder of the subject as a distracter. The distracter receives 75% of the stimuli, while the left and right wrist each receives 12.5% of the stimuli. Then, the subject is instructed through earplugs to count stimuli to the target hand (15 targets, 7^*^15 non-targets), which is either the left or right hand. During each run, the subject performs this task four times, with the target hand selected randomly each time, resulting in a recording time of 2.5 min. The VT2 and VT3 paradigms are jointly called the EP paradigms, as they rely on evoked potentials (EPs) such as the P300.
3. Motor imagery (MI): In this paradigm, the subject is verbally instructed to imagine a left or right hand movement (chosen randomly) for 4 s. Each such period is followed by a random interval of 0.5–2 s to avoid adaptation before the next instruction. The run lasts 9 min and records 30 imagined movements of each hand.

#### Signal processing and classification

During the assessment runs, the raw EEG data and the stimulation time points are recorded, and this data is used to calibrate the BCI for every subject. In the VT2 and VT3 paradigms, data segments of −100 to 600 ms around each stimulus are extracted and the data is classified using linear discriminant analysis (LDA) to distinguish target from non-target stimuli. This results in a classification accuracy ranging from 0 to 100% that describes how well the data can be separated. The ratio of target to non-target stimuli is 1: 7, which results in a chance accuracy of 12.5%. In the MI BCI system, the data (filtered with 8–30 Hz) from each imagined movement is used to train a common spatial patterns (CSP) algorithm that automatically weights each electrode according to its contribution to discrimination accuracy. In this case, the data from the window 3–5 s after the instruction is used to train the CSP algorithm. Next, the variance of a 1.5 s window is estimated and an LDA classifier is trained to calibrate the system on the subject (Guger et al., 2000). This results in a classification accuracy ranging from 0 to 100% with a chance accuracy level of 50% (2-classes are discriminated). The calibration data is saved for future communication tests, which are described in the following section. In MI mode the classification accuracy is calculated with a 10 times 10-fold cross-validation to have independent training and testing data. In VT2 and VT3 mode the data is randomly shuffled and 50% are used for training and 50% are used for testing and this procedure is repeated 10 times.

In addition to classification accuracy, mindBEAGLE also calculates EPs for VT2 and VT3 to compare target and non-target stimuli. The data is extracted from −100 to 600 ms around the stimulation, baseline corrected and averaged. A significance test is performed that presents areas with significant differences between targets and non-targets as green-shaded areas in the EPs (*p* < 0.05). Trials where the amplitude of the EEG signal exceeds ±100 μV are rejected from the EP and classifier calculation.

#### Communication

A communication test can be performed with VT3 and MI. In the VT3 paradigm, the operator can ask the subject a question, and the subject can answer either YES or NO by counting the stimuli on either the left or right hand. It is necessary to ask questions to which the answers are known (called copy-spelling mode in the BCI literature, e.g., Are you born in Italy?) to evaluate the system accuracy. In the VT3 paradigm, one question can be answered after 120 stimuli, which requires 38 s. The system only selects YES or NO if the result is significant. In the MI paradigm, the subject can answer either YES or NO by imagining either left or right hand movement for 8 s. In the MI mode the system will always make a YES or NO decision. In both paradigms, the calibration data from the previous assessment run is used to calculate a subject specific classifier that is used for the communication test. During the task no feedback is provided to the participant, but after the questions the answer is reported to the patient. During the communication the subject specific classifier is not updated.

### Participants and recording locations

Table 1 presents the two locations where data were recorded for this study: Schiedlberg and Palermo. Each of these locations had ethical approval for this study from the appropriate supervisory board at that location (Ethic committee Palermo 1 from the University Hospital Palermo and Karl Franzens University Graz). Written informed consent was obtained from all research participants or legal guardians. Notably, all patient data were recorded at each patient's bedside by medical doctors who did not have an engineering background, without support from any technical staff. The doctors were trained beforehand to operate the system and were shown how to check the EEG signal.

**Table 1:** **Recording locations**.

**Location**	**Name of institute**	**ID**
Palermo	University of Palermo, Italy	PA
Schiedlberg	Guger Technologies OG, Austria	GT

This paper presents results from 12 patients (3 CLIS and 9 LIS) and 3 healthy controls. We recruited 12 patients with a diagnosis of Amyotrophic Lateral Sclerosis according to the El Escorial diagnostic criteria, regularly followed-up in the ALS Clinical Research Center and in a LIS or CLIS state.

Details are shown in Table 2. These data were selected to show the following outcomes, including both assessment and communication:

**Table 2:** **Overview of healthy controls and patients participating in this study**.

**#**	**Sex**	**Age (years)**	**Diagnosis**	**Disease duration (month)**	**Mechanical ventilation**	**Clinical state**	**Rec. site**
**HEALTHY CONTROLS**							
S1	F	42	Healthy	–	–	–	GT
S2	M	43	Healthy	–	–	–	GT
S3	M	38	Healthy	–	–	–	GT
**PATIENTS**							
P1	F	61	ALS	149	yes	CLIS	PA
P2	M	67	ALS	97	yes	LIS	PA
P3	F	76	ALS	145	no	LIS	PA
P4	F	75	ALS	184	yes	CLIS	PA
P5	F	68	ALS	89	yes	LIS	PA
P6	M	63	ALS	27	yes	LIS	PA
P7	F	62	ALS	70	yes	CLIS	PA
P8	M	68	ALS	52	yes	LIS	PA
P9	F	65	ALS	84	no	LIS	PA
P10	M	37	ALS	103	yes	LIS	PA
P11	M	58	ALSFTD	21	yes	LIS	PA
P12	F	46	ALS	136	yes	LIS	PA

“ALSFTD” means “ALS with frontotemporal dementia.”

-VT2, VT3, and MI results for three healthy controls, in both regular system operation and sham conditions; and -VT2, VT3, and MI results for LIS patients, in regular system operation.

### Experimental procedure

#### LIS participants' procedure

Informed consent was obtained prior to the assessment. Since most participants were unable to provide consent due to their disabilities, informed consent was obtained from the patient's legal guardian. Patients were lying or sitting in bed during the assessment and the medical institution selected pseudo-randomly one of the available paradigms, but VT2 always before VT3. If the classification accuracy was higher than 60% for the VT3 (well above the 95% confidence interval with a binomial test that gives about 23%) and higher than 63.19% (95% confidence interval for 60 trials using a binomial test) for motor imagery, the staff may have attempted communication. The decision of whether to attempt communication included considerations such as the physician's assessment of the patient's fatigue and available time; thus, clinical considerations were prioritized over research needs.

#### Healthy participants and sham procedure

The healthy participants gave informed consent directly, without a legal guardian, and were seated. The experimental protocol was similar to the LIS patients, with the following changes. The healthy participants performed two regular runs and one sham run, across the VT2, VT3, and MI assessment paradigms, and then across the VT3 and MI communication paradigms. The order of these runs was determined pseudo-randomly, and there were nine total assessment runs and four total communication runs. The communication runs each entailed five or ten yes/no questions. During the VT2 sham runs, the vibrotactile stimulators were unplugged. Thus, while subjects still wore the cap and heard instructions during the VT2 sham runs, they never received tactile stimuli required to elicit EPs. During the VT3 and MI sham runs, the system was muted. Thus, during these two types of sham runs, the patients wore the cap, but did not hear instructions that were essential for normal system operation.

This sham assessment was conducted to confirm correct system operation. We hypothesized that the healthy participants would exhibit robust EP differences and thus high classification accuracy in the regular (and not sham) EP paradigms. Indeed, any other outcome would raise serious concerns about the other results presented here. We also expected that the healthy participants would exhibit left vs. right hand imagery differences in the regular (and not sham) MI runs, although untrained healthy users can exhibit variable performance with MI BCIs.

## Results

### Healthy controls and sham results

Table 3 summarizes results from the regular and sham runs with the three healthy subjects. The table includes classification accuracies during assessment runs and communication. For the two EP-based approaches (VT2 and VT3), accuracy is between 60 and 100% in regular runs, and 0 to 25% for sham runs. For the MI approach, accuracy is above the significance threshold (63.19%) during the two regular runs, and near chance level (below the significance level) for the sham run. The column communication indicates that the accuracy of the communication mode ranged from 20 to 100%. For assessment, the mean accuracy of VT2 was higher than the mean accuracy of VT3. VT3 communication was slightly more accurate than MI communication.

**Table 3:** **Classification accuracies during assessment and communication runs, and number of questions answered correctly (e.g., 5 questions are answered correctly out of 5), from subjects S1–S3 (healthy)**.

**Subject**		**S1**		**S2**		**S3**		**Total**	
	**Exp**.	**Ass. Acc. [%]**	**Comm. [%]; [correct/total]**	**Ass. Acc. [%]**	**Comm. [%]; correct/total]**	**Ass. Acc. [%]**	**Comm. [%]; correct/total]**	**Mean ass. accuracy [%]**	**Mean commu. Acc. [%]**
VT2	Run 1	100		100		65		88	
	Run 2	100		100		100		100	
	Sham	0		25		10		12	
VT3	Run 1	90	100, [5/5]	60	20, [1/5]	100	100, [5/5]	83	73.3
	Run 2	100	80, [4/5]	100	100, [5/5]	80	80, [4/5]	93	86.7
	Sham	0		10		10		6.6	
MI	Run 1	70	80, [4/5]	71	60, [3/5]	78	80, [4/5]	73	73.3
	Run 2	90	80, [4/5]	67	80, [4/5]	62	90, [9/10]	73	83.3
	Sham	55		50		59		57	

*Chance accuracy was 12.5% in both EP runs and 50% in the MI runs. As an example 4/5 means that 4 answers out of 5 questions were given correctly and 1 answer was either undetermined or wrong in VT3 communication mode and wrong in MI communication mode. The VT2 and VT3 assessment runs last 2.5 min, the MI assessment runs lasts 9 min. In VT2 and VT3 communication it takes 38 s to answer 1 question and in MI communication it takes 8 s to answer 1 question*.

**Figure 2:**
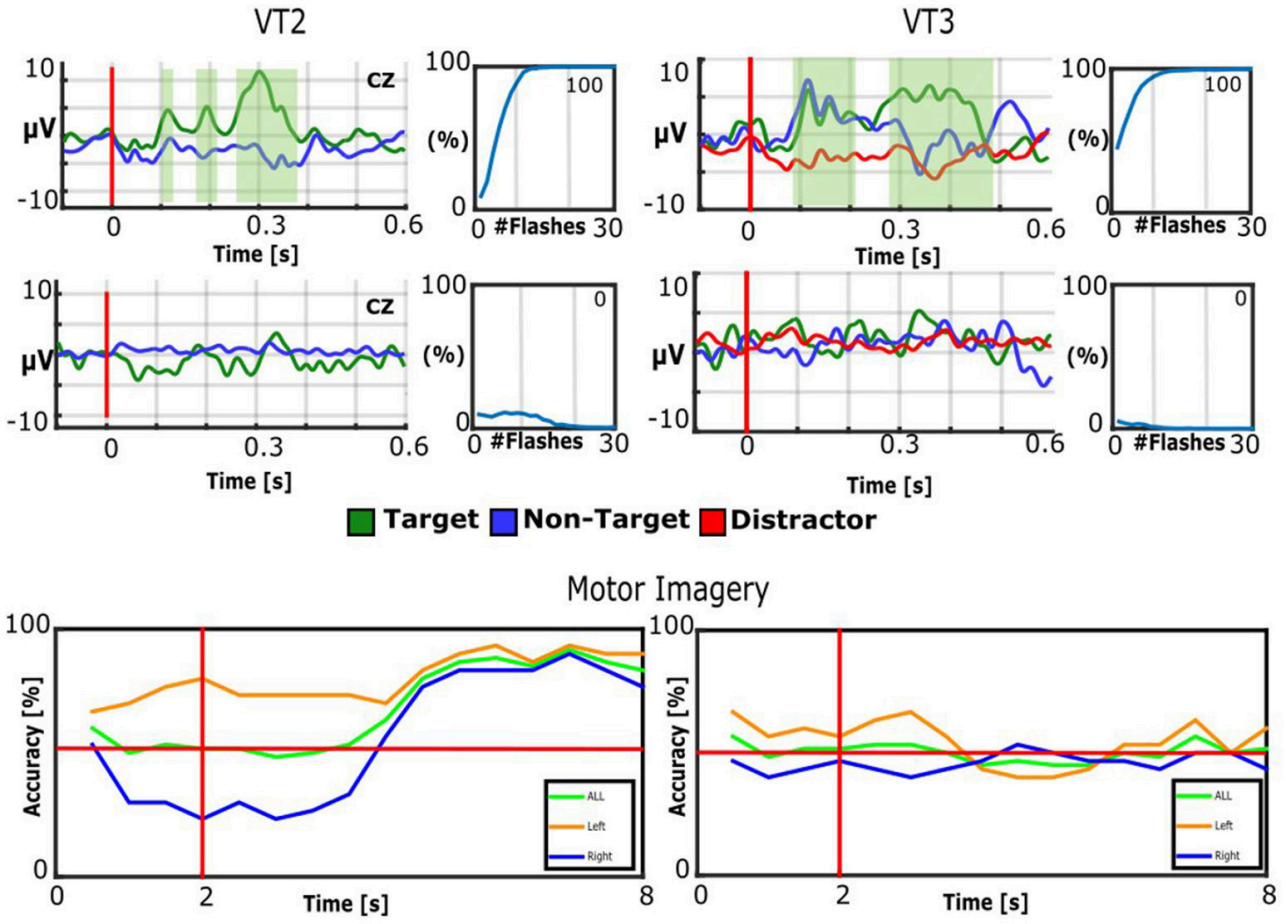
**Results of healthy control subject S1**. Top: EPs of Cz and BCI accuracy for VT2 and VT3. The EP plots contain target EPs in green and non-target EPs in blue. The VT3 EP plots also contain the distractor in red. The green shaded areas indicate significant differences between targets and non-targets. The BCI accuracy is plotted over number of target stimuli. 12.5% would be chance level. Middle: sham runs. The number presented in the top right of each accuracy plot is the mean accuracy. Bottom: The BCI accuracy over time. The vertical red line indicates when the cue is presented to the subject, while the horizontal red line represents 50% accuracy (corresponding to chance performance). The plot shows the results averaged for all trials of left hand imagination, right hand imagination and imagination of both hands on the left and the results for sham on the right.

Figure 2 presents results from one healthy control (S1) for all three paradigms (VT2, VT3, and MI) for one regular run and one sham run. In the VT2 and VT3 paradigms, the subject showed a robust P300 response to target events only, and attained 100/100% classification accuracy within less than 12 stimulus repetitions. As expected, during sham runs, the subject does not exhibit clear target vs. non-target EP differences, and the accuracy is below 12.5% (chance accuracy). Please note that the VT2 paradigm had seven times more non-target trials than target trials, which explains why the non-target EPs look smoother than the target EPs. In the VT3 paradigm the distractor had more trials than the target and non-target. In the MI paradigm, the mean accuracy from second 5 to 7 is 86.3% for run 2 and 49.0% for the sham condition.

### Patients

Table 4 shows the results over all patients for VT2, VT3 and MI paradigms, including assessment and communication results. For assessment, VT2 results are between 20 and 100%, VT3 is between 10 and 100% and MI is between 42 and 83%. In VT3, all communication tests resulted in correct answers to at least 7 out of 10 questions—8 out of 10 on average. If the VT3 assessment reached 100% accuracy (in 5 runs), then the communication was between 80 and 90% accurate. MI communication was successful in all communication tests (3 runs).

**Table 4:** **Results from 12 patients. Median classification accuracies are shown for VT2, VT3 and MI assessment sessions**.

**Pat**.	**Sess. #**	**VT2, 4 instructions [%]**	**VT3, 4 instructions [%]**	**VT3 Com**	**MI, 60 instructions [%]**	**MI Com**	**Total recording time [min]**
P1	1	100	100	9/10	51	–	<15
P2	1	100	70	7/10	73	4/5	<15
P3	1	100	90	8/10	59	–	<15
P4	1	20	70	7/10	47	–	<15
P5	1	99	100	9/10	83	5/5	<15
P6	1	80	100	9/10	56	–	<15
P7	1	40	40	–	–	–	<10
	2	–	20	–	–		
P8	1	70	100	8/10	49	–	<15
P9	1	40	10	–	–	–	5
P10	1	100	50	–	54	–	<20
	1	–	90	8/10	–	–	
P11	1	70	20	–	52	–	<10
	1	–	20	–	–	–	
P12	1	100	60	–	42	–	<10
	1	–	70	7/10	74	5/5	
Average		76.6	63.1	8/10	58.2	4.7/5	

*Communication accuracy is also presented as the number of questions answered correctly out of 10 questions (VT3) or 5 questions (MI). As an example 9/10 means that 9 answers out of 10 questions were given correctly and 1 answer was either undetermined or wrong in VT3 communication mode and wrong in MI communication mode. Runs are shown in different rows for a session. A “-“ shows that the paradigm was not performed. The VT2 and VT3 assessment runs last 2.5 min (4 instructions with 15 targets each), the MI assessment runs lasts 9 min (60 instructions). In VT2 and VT3 communication it takes 38 s to answer 1 question and in MI communication it takes 8 s to answer 1 question*.

**Figure 3:**
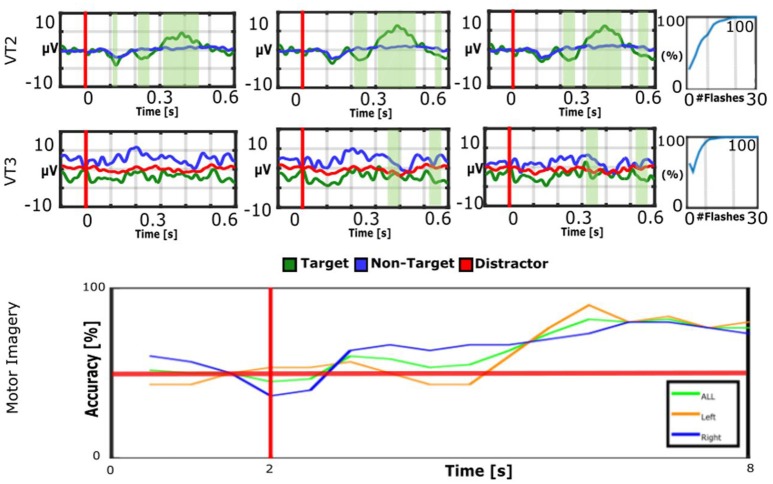
**EPs and accuracies for VT2, VT3, and MI runs of LIS patient 5**. The patient achieved an average accuracy of 83% for MI, 100% for VT2, and 100% for VT3. Communication was possible with the MI and VT3 paradigms. In the bottom figure, the vertical red line indicates when the cue is presented to the subject, while the horizontal red line represents 50% accuracy (corresponding to chance performance).

Figure 3 shows VT2, VT3, and MI results from LIS patient 5. The VT2 EP shows significant target vs. non-target differences, and the P300 is prominent around 300–500 ms. For the VT3, the EP differences are less prominent to the naked eye. The median accuracy for VT2 is 100%, and for VT3, it is 100%. The classifier is able to separate the motor imagery data with 83% accuracy.

**Figure 4:**
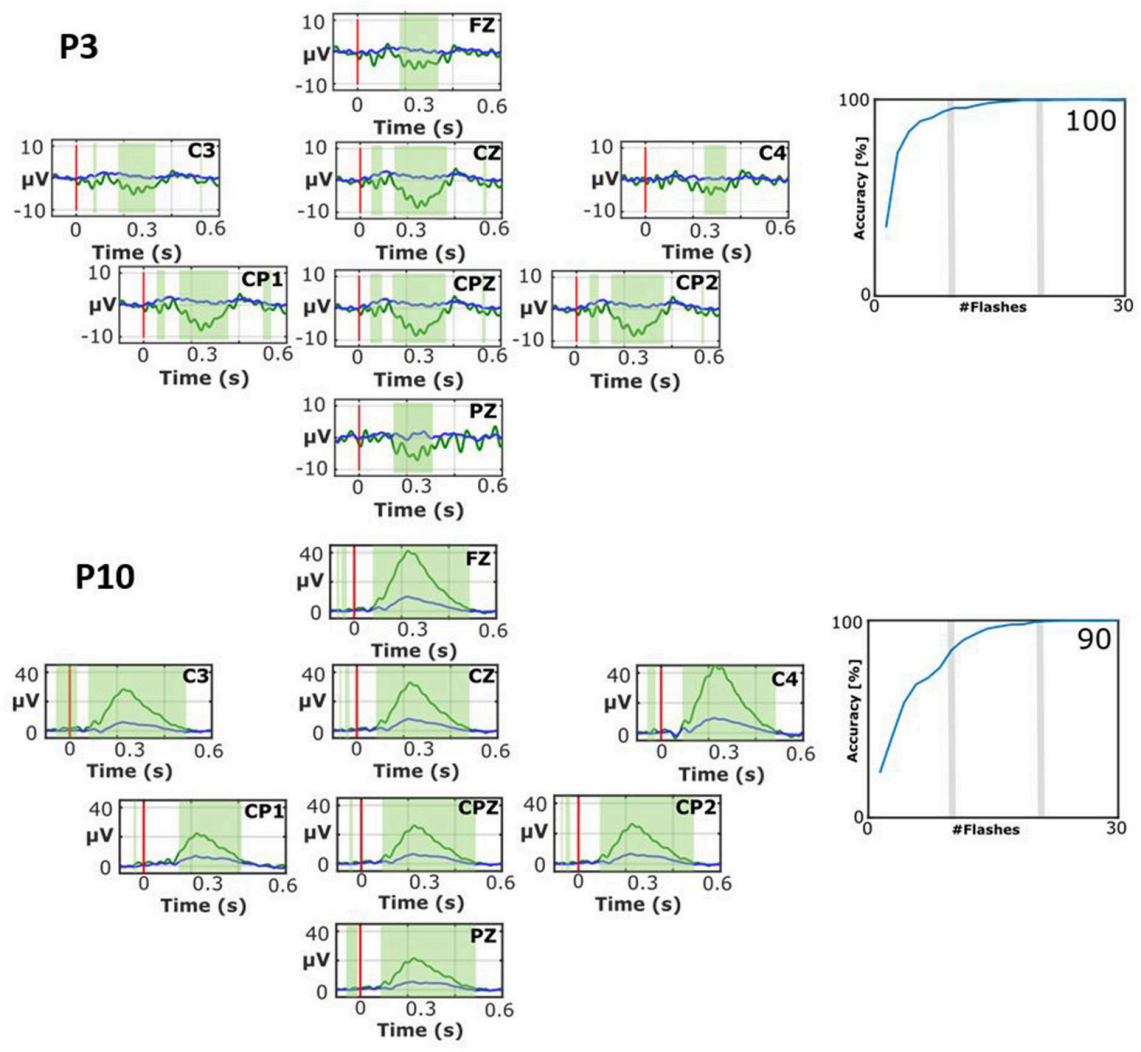
**EPs and classification accuracies for patients P3 during the VT2 assessment paradigm (top) and P10 during the VT3 assessment paradigm (bottom)**. The number presented in the bottom right of each accuracy plot is the mean accuracy. While both patients attained high accuracies, their EPs exhibit atypical waveforms.

Figure 4 shows two interesting cases with atypical EPs. P3 shows a broad negative peak to target stimuli only that is largest around 250 ms on all 8 channels that are analyzed for the VT3 paradigm used here. The EPs do not exhibit typical peaks such as the N1, P2, N2, or P3. Nonetheless, the classification accuracy reached very soon 100% accuracy (median accuracy 100%), which shows that the target vs. non-target EPs can be separated despite their unusual waveforms.

P10 also shows an atypical waveform. P10 has an early positive wave to target stimuli that peaks a little after 200 ms. This signal has a peak amplitude of 42.5 μV on C4, and is robust on other sites as well. The non-target stimuli produced a much smaller positive peak at about the same time. The waveform preceding this positive peak has very small inflections that look similar to peaks that precede the P300, but these peaks (like the large positive peak) seem too early. The median classification accuracy is 90%, which shows the clear difference between targets and non-targets.

## Discussion

The VT2, VT3, and MI assessment paradigms were used in healthy persons and LIS/CLIS patients. All healthy controls and 10 out of 12 LIS patients were able to achieve high accuracies during the assessment runs (≥70%). 9 out of 12 LIS patients also communicated with the VT3 paradigm, and 3 of these also communicated with the MI paradigm. More importantly the system allowed 2 CLIS patients out of 3 to communicate. This was not shown beforehand with an EEG-based BCI system (Chaudhary et al., 2017). The VT2 paradigm (76.6%) allowed a higher mean accuracy than VT3 (63.1%), which was more accurate than MI (58.2%). There are several reasons for these accuracy differences. First, the VT2 and VT3 assessment paradigms are only 2.5 min long, instead of 9 min for the MI assessment paradigm, and therefore it may be easier for patients to maintain concentration. Second, the VT2 and VT3 assessment paradigms have more stimuli per second than the MI assessment paradigm, which may also help patients maintain concentration. Third, the VT2 paradigm is the easiest for patients. Unlike the other two paradigms, it is a passive paradigm, and can yield EP differences to target vs. non-target stimuli even if the patient is not actively participating. In this study, the patients were instructed to actively count the target (deviant) stimuli to produce an active P300 response that is normally higher than a passive P300 response. Also, the VT3 paradigm exhibits training effects, presumably because it is slightly harder to learn (Ortner et al., 2013). Fourth, the literature has shown that some training is required to attain good accuracy with MI BCIs, and subjects in this study were untrained. Fifth, the literature has also shown that some persons are never able to attain high accuracy with MI BCIs, which seems to be less problematic with P300 BCIs (Allison and Neuper, 2010; Acqualagna et al., 2016).

A recent study achieved a mean accuracy of 80% with VT2 and 55.3% with VT3 in 6 LIS patients. This is a bit better than in this study for VT2 (+3.4%) and a bit worse for VT3 (−2,9%) but did not record from CLIS (Lugo et al., 2014). The current study has a mean accuracy of 80% for VT2 and 57.5% for VT3.

The advantage of the EP assessment paradigms is that both can be used very quickly to see if the patient is able to perform the task as shown step-by-step in (Ortner et al., in press). They can also be used to quickly determine whether the patient is awake or sleeping during the BCI experiment. 9 of the 12 patients were able to communicate effectively using the VT3 paradigm. Two of these patients (P10 and P12) needed an additional assessment run to achieve a high enough classification accuracy of >60%, while 7 patients achieved high enough classification accuracy in the first run. P7 and P9 achieved only 10−50% accuracy across both types of EP runs, which was too low for communication. P11 achieved 70% with the VT2 run, but this mode does not allow communication. In the VT3 mode, P11's accuracy was too low for communication. This result with P11 may indicate that P11 was able to perform a passive paradigm such as VT2, but not an active paradigm like VT3. P11 was the only person in this study diagnosed with frontotemporal dementia, which may explain this result (see Table 2). Another reason could be that the patient just needs more training time or that the patient was already tired after the VT2 run.

If the VT3 assessment accuracy was above 60%, then a communication run was also performed, in which 10 questions with a known answer were asked. No patient was able to answer all questions successfully, but 3 patients answered 9 correctly, 3 answered 8 correctly, and 3 answered 7 correctly. The healthy persons during the non-sham runs attained an overall mean communication accuracy of about 80%. Notably, 1 healthy person (S2) achieved only 20% accuracy in the first VT3 run and was able to improve to 100% in the second run. This result with S2 supports our supposition that the VT3 paradigm may be more difficult than VT2 and entail some training, which was also observed in both P10 and P12. The successful communication in the study showed that the 60% threshold could eventually also be lowered in the future to establish communication.

In MI mode, 3 patients' MI assessments indicated the potential for communication, and all three of them successfully communicated. The assessment accuracies were only 73, 83, and 74%, but they were able to answer 14 out of 15 questions correctly. The MI classifier is calculated from 60 right and left motor imagery trials that each entail 4 s of motor imagery. The communication mode provides 8 s to answer each question, which provides more data for analysis and results in the higher communication accuracy compared to the assessment accuracy. For the healthy controls, the mean MI assessment accuracy was about 71%, but 33 out of 40 questions were answered correctly (82.5%).

It is also important to note that participant did not get any feedback during the assessment or communication runs while the experimenter was able to see the on-line updated evoked potentials of all 8 channels or the on-line updated ERD maps. After a question was answered with the system, the experimenter communicated the selected answer to the participant. This could have an important motivation effect for the patient, but we don't have enough data to show any training effects.

The system is using 8 EEG channels for the VT2 and VT3 paradigms and allows us to see which regions are generating EPs. 8 EEG channels are also used for visual P300 spellers, which yielded a grand average accuracy of 100% for a 17 healthy subjects group study (Guger et al., 2016a). The motor imagery paradigm uses 16 channels to increase classification accuracy with the CSP algorithm. With healthy controls, an accuracy of around 80% can be expected with minimal training (Ortner et al., 2015). Ortner and colleagues used 64 channels for this study, but similar accuracies can also be achieved with fewer electrodes with a dense arrangement around the sensorimotor cortex (Guger et al., 2000).

The BCI technology makes it easy to determine whether target and non-target stimuli can be discriminated, which is sometimes difficult if the EP waveform is only investigated visually. Visual inspection of averaged EPs may miss trial to trial variations in EPs, and the BCI system takes every single trial into consideration. The BCI system also indicates how many stimuli are required to reach different levels of classification accuracy, which reflects the difference in P300 s and other EPs resulting from attention to the target stimulus. More robust EP differences will lead to a higher accuracy earlier than weaker responses.

An important feature of the system is that different paradigms can be applied for assessment and communication. A recent study used fMRI and motor imagery EEG to detect command following and therefore awareness in VS and MCS patients. 3 out of 6 patients showed spatial navigation with fMRI, 1 out of 6 showed motor imagery with fMRI and 2 out of 6 showed motor imagery with EEG (Gibson et al., 2014). Also, in the current study, only 3 out of 12 patients showed successful communication with the motor imagery BCI, but 9 out of 12 were able to use the VT paradigm successfully. Therefore, a system with standardized tests is highly recommended for testing patients. A further advantage of the current system compared to fMRI is that it is easy to use, fast to use, portable, and of low cost (Guger et al., 2016b).

Another study recently published showed that an fNIRS based system is able to establish yes/no communication with CLIS (Chaudhary et al., 2017). These patients were trained to regulate their frontal centered brain regions in response to auditory questions and were trained in more than 40 sessions to reach accuracies between 64.3 and 78.8% in LIS and CLIS. In the current study the grand average communication accuracy was 80% for all patients that were able to communicate and this is clearly better than with fNIRS. The current study also includes all patients that were available at the clinical center. A further advantage of the current system is the short assessment and communication time. All patients were only trained for 5 to 20 min and communication was established in the majority. With additional training even a higher success rate can be expected.

A limitation of the study is the lack of time to perform more training. It could be that patients that did not communicate in this study could do that with more training time. Also, several runs could be done on different days to describe training effects and fluctuations.

In the study we enrolled all LIS/CLIS patients that were available for the University of Palermo. Three patients were diagnosed as CLIS and 9 patients were LIS and the study could show that a majority of patients can communicate with the system.

In the future, we will further implement these tests with VS/USW and MSC patients to study the absence/presence of vibro-tactile P300 responses and motor imagery responses as diagnostic tools. In these patients, the EEG has many advantages compared to fMRI recordings: (i) easy to use, (ii) inexpensive, (iii) works with patients with metallic implants, (iv) faster brain response.

## Summary

While earlier results have shown that persons with LIS resulting from ALS can communicate with BCIs that do not rely on visual stimuli, the present study extends these results in three ways. First, this is the first study that shows that an EEG-based BCI system can establish communication with CLIS. Second, it shows that non-visual BCI technology can be used to assess command following in these patients. Third, this is the first study to show that the mindBEAGLE system, which is designed to be easy to use without technical expertise, can provide a practical and usable platform for both assessment and communication in these patients. This outcome is reflected in the right panel of Figure 1.

Healthy controls achieve higher accuracies in assessment and communication than LIS patients, but 9 out of 12 LIS patients could use the VT3 paradigm to answer questions. Only 3 out of 12 LIS patients could use the MI based system to answer questions after a training run. Therefore, the VT3 paradigm can provide limited communication with a LIS patient without substantial training. Although the MI paradigm could not provide communication for most subjects, it can potentially provide faster communication than the VT3 paradigm. Like most BCIs, the trade-off between speed and accuracy is largely a function of each user's abilities and preferences; the MI and VT3 paradigms could be faster at the expense of reduced accuracy, and vice versa.

A big advantage of a flexible assessment and communication system is the ability to quickly test whether a patient is able to answer questions, which can provide insight about remaining cognitive functions. In future the mindBEAGLE system will also be tested to identify fluctuations of awareness in order to guide the usage of other BCI-based platforms for communication and restoration of function.

## Author contributions

RS was primarily responsible for collecting the data from the patients, which occurred in collaboration with VL. The remaining authors were responsible for conceptual and technical development of mindBEAGLE, data analysis, study design, and/or manuscript preparation.

## Funding

This work was supported by EC H2020 project ComaWare.

### Conflict of interest statement

CG is the co-CEO of the two entities listed in the first two author affiliations. AH, RO, BA, and WC are all employed by one of these two entities. The other authors declare that the research was conducted in the absence of any commercial or financial relationships that could be construed as a potential conflict of interest.
